# Assessment of esophageal involvement in systemic sclerosis and morphea (localized scleroderma) by clinical, endoscopic, manometric and pH metric features: a prospective comparative hospital based study

**DOI:** 10.1186/s12876-015-0241-2

**Published:** 2015-02-15

**Authors:** Tasleem Arif, Qazi Masood, Jaswinder Singh, Iffat Hassan

**Affiliations:** 1 Postgraduate Department of Dermatology, STDs & Leprosy, Government Medical College, Srinagar, Jammu and Kashmir India; 2 Department of Gastroenterology, SKIMS, Soura, Srinagar, Kashmir India; 3 Postgraduate Department of Dermatology, STDs and Leprosy, Jawaharlal Nehru Medical College (JNMC), Aligarh Muslim University (AMU), Aligarh, India

**Keywords:** Endoscopy, Esophageal manometry, Morphea, Reflux esophagitis, Systemic sclerosis

## Abstract

**Background:**

Systemic sclerosis (SSc) is a generalized disorder of unknown etiology affecting the connective tissue of the body. It affects the skin and various internal organs. Gastrointestinal tract involvement is seen in almost 90% of the patients. Esophagus is the most frequently affected part of the gastrointestinal tract. Esophageal motility disturbance classically manifests as a reduced lower esophageal sphincter pressure (LESP) and loss of distal esophageal body peristalsis. Consequently, SSc patients may be complicated by erosive esophagitis and eventually by Barrett’s esophagus and esophageal adenocarcinoma. Morphea, also known as localized scleroderma, is characterized by predominant skin involvement, with occasional involvement of subjacent muscles and usually sparing the internal organs. The involvement of esophagus in morphea has been studied very scarcely. The proposed study will investigate the esophageal involvement in the two forms of scleroderma (systemic and localized), compare the same and address any need of upper gastrointestinal evaluation in morphea (localized scleroderma) patients.

**Methods:**

56 and 31 newly and already diagnosed cases of SSc and morphea respectively were taken up for the study. All the patients were inquired about the dyspeptic symptoms (heartburn and/or acid regurgitation and/or dysphagia). Upper gastrointestinal endoscopy, esophageal manometry and 24-hour pH monitoring were done in 52, 47 and 41 patients of SSc; and 28, 25 and 20 patients of morphea respectively.

**Results:**

Esophageal symptoms were present in 39 cases (69.6%) of SSc which were mild in 22 (39.3%), moderate in 14 (25%), severe in three (5.3%); while only four cases (7.1%) of morphea had esophageal symptoms all of which were mild in severity. Reflux esophagitis was seen in 17 cases (32.7%) of SSc and only two cases (7.14%) of morphea. Manometric abnormalities were seen in 32 cases (68.1%) of SSc and none in morphea. Ambulatory 24-hour esophageal pH monitoring documented abnormal reflux in 33 cases (80.5%) of SSc and no such abnormality in morphea.

**Conclusion:**

While the esophageal involvement is frequent in SSc, no such motility disorder is seen in morphea. Meticulous upper gastrointestinal tract evaluation is justified only in SSc and not in morphea.

## Background

Systemic sclerosis (SSc) is a generalized disorder of unknown etiology affecting the connective tissue of the body. It affects the skin and various internal organs like gastrointestinal tract, lungs, heart and kidneys [1]. Gastrointestinal tract involvement is very common, affecting about 90% of the systemic sclerosis patients [2,3]. Esophagus is the most frequently affected part of the gastrointestinal tract [4]. Esophageal smooth muscle becomes atrophied and replaced by fibrous tissue leading to severe motility disturbance of distal esophagus [5,6]. Esophageal motility disturbance classically manifests as a reduced lower esophageal sphincter pressure (LESP) and loss of distal esophageal Body peristalsis [7-9]. As a consequence of this involvement, patients usually manifest with heartburn, dysphagia and regurgitation [10]. Heartburn and regurgitation are due to reflux of gastric juice across an incompetent lower esophageal sphincter (LES), whereas dysphagia may result from esophageal peptic stricture or disturbed esophageal peristalsis [11,12]. Esophageal complications like esophageal stenosis, Barrett esophagus and esophageal adenocarcinoma are more frequent in SSc than the general population [4,13-17].

Morphea, also called as localized scleroderma, predominantly involves the skin and occasionally involves subjacent muscles. However, it usually spares the internal organs. Morphea may range from small plaques to extensive disease with cosmetic and functional deformities [18]. The esophageal involvement in morphea has been studied scarcely and the data regarding this subject is meager. The present study was designed to investigate the esophageal involvement in the systemic (SSc) and localized (morphea) forms of scleroderma and to compare the same. It will also address any need of upper gastrointestinal evaluation in the morphea (localized scleroderma) patients.

## Methods

This was a hospital based study carried out in the Postgraduate Department of Dermatology, Sexually Transmitted Diseases and Leprosy of Shri Maharaja Hari Singh (SMHS) Hospital (Associated teaching hospital of Government Medical College Srinagar) and the Department of Gastroenterology Sheri-Kashmir Institute of Medical Science (SKIMS) Soura. It was a prospective observational study involving the newly as well as already diagnosed patients of systemic sclerosis and morphea over a period of one and a half year (March 2011-August 2013).The study was approved by the ethical committees of the two hospitals viz., Institutional Ethics Committee (IEC) SKIMS and Ethical committee Government Medical College (EC-GMC) Srinagar. The diagnosis of systemic sclerosis was made according to the American Rheumatology Association (ARA) criteria [19]. Morphea was diagnosed by the clinical and histopathological features after taking a standard punch biopsy of the skin.

Inclusion criteria: 1) All newly as well as already diagnosed patients of systemic sclerosis and morphea. 2) Both sexes were included. 3) Age ≥ 13 years.

Exclusion criteria: 1) Presence of pregnancy or a history of pregnancy in the last six months. 2) Age <13 years. 3) Other connective tissue disease or mixed connective tissue diseases. 4) Diabetes mellitus.

In the primary assessment, data collected included patient’s age, gender, clinical characteristics of the disease (age at onset, duration), type of systemic sclerosis (diffuse or limited defined according to Le Roy classification [20]) and the presence or absence of symptoms of gastro-esophageal reflux disease (GERD) viz., heartburn, acid regurgitation and dysphagia. Each symptom was graded on a scale from 0 to 3 by intensity (0 = absent, 1 = mild, could be ignored by the patient, 2 = moderate, could not be ignored, but had no effect on daily life activities; 3 = severe or incapacitating, affecting daily life activities) and by frequency (0 = absent or less than one per month; 1 = less than 1 per week; 2 = several times per week; 3 = every day) [21]. Symptoms were then categorized as mild (score less than or equal to six), moderate (score of seven to twelve) and severe [score greater than twelve, or when one symptom was considered incapacitating every day (score = 9)]. Drugs which are known to suppress acid (Proton pump inhibitors and H2 blockers) or alter esophageal motility (anticholinergics, sedatives, antihypertensive and anti-angina drugs) were discontinued 2 weeks before inclusion. A proper consent (verbal and written) was given by the patient or his guardian before carrying out any procedure, for the participation in the study and for the consequent publication of the data which may also contain their personal details and their images. The patients were enrolled in the study only after meeting the above requirements of the consent.

### Esophago-gastroduodenoscopy (EGD)

52 patients (out of total 56) of SSc and 28 patients (out of total 31) of morphea were undertaken for upper gastrointestinal endoscopy. Fibreoptic video-endoscope (Fujinon, EG-201FP, Japan) was used to look for the signs of esophagitis which was graded according to the Los Angeles classification for reflux esophagitis [22,23].

### Esophageal manometry

This procedure was performed to measure lower esophageal sphincter pressure and amplitude of the body contractions of distal esophagus. Patients were instructed to wear loose clothes and avoid wearing necklace. The procedure was conducted in the supine position with the patient fasting over night. The manometric instrument used in our study (Red Tech, inc 26234 Alizaa Cnayon Dr. Los Angeles, USA 91302) consisted of a special mutilumen (16 channel) catheter system. The catheter was connected to external pressure transducers. The catheter was continuously perfused with distilled water at a rate of 0.5 ml/min by a low compliance pneumohydraulic capillary infusion system. The catheter assembly was passed through the nose after applying xylocaine jelly locally until all recording orifices were in the stomach. The station pull-through of the lower esophageal sphincter (LES) was performed at one cm intervals. The LES pressure recorded was measured at end-expiratory variation to the mean gastric baseline pressure. At least, 10 wet swallows (10 ml water each) were administered; each separated by 30 seconds period. The amplitude of pressure wave was measured from the mean intraesophageal baseline pressure to the peak of the wave. Reference values for esophageal manometry were taken from Benjamin et al. [24].

### Ambulatory 24-hour esophageal pH monitoring

This procedure was performed to objectively document abnormal reflux of gastric acidic contents into the lower esophagus and the consequent drop in lower esophageal pH. It was done after a standard esophageal motility study. Lower esophageal pH was measured with an esophageal probe (Antimony probe). The pH electrode was passed through the anesthetized nose of sitting patient into the stomach until acid pH was recorded. After that, the patient would remain supine and electrode was slowly withdrawn in the supine position. In each case a rapid pH change from acid to above pH 5 could be identified and pH electrode kept 5cms above this identified Zone (LES) already determined by manometric technique. The distance between the tip of the catheter and the nostril was recorded and kept constant for 24-hour esophageal pH study. The pH measuring unit was calibrated at 37°c using buffer solution of pH 4 and 7. The pH probe and reference electrodes were connected to a portable solid state recorder (Red Tech Medical Systems Pvt. Ltd) which is a family of portable self programmable data loggers for recording the biological variables completely based on micro processing technology. The esophageal pH measurements were stored and then transferred to a computer for analysis. The equipment used by us for pH manometry was an older version lacking the option for calculating the impedance-pH metry which is currently considered to be the gold standard for studying the gastroesophageal reflux disease. Reflux disease was considered abnormal if any of the following criteria were exceeded: 1) Percentage of total time with pH <4 (normal <5.5%); 2) Percentage of upright time with pH <4 (normal <8.2%); 3) Percentage of supine time with pH <4 (normal <3%) [8]; 4) De-Meesters Score (normal <14.7) [25]. Patients with abnormal reflux were considered as refluxers; those with upright reflux were classified as mild refluxers, supine as moderate and combined as having severe reflux.

### Statistical analysis

The data collected was analyzed by using statistical package for social sciences (SPSS) Version 16.0. The following tests were also used: Chi-square test**,** Fischer’s Exact test and Student’s ‘t’ test. A p value of < 0.05 was considered as statistically significant.

## Results

Fifty six patients of SSc and 31 patients of morphea were taken up for the study. Among SSc patients, 50 (89.3%) were females and 6 (10.7%) were males with a female to male ratio of 8.3:1. The average age of the patient was 44.96 ± 13.80 years (21–80). Most of the patients 17 (30.4%) were in the age group of 50–59, followed by 40–49 (12, 21.4%) (Table 1). The average age of the onset of the disease in SSc in case of females was earlier (35.2 ± 13.3 years) than in males (42.3 ± 13.5 years) but it was statistically insignificant (p = 0.219). However, the average duration of disease in case of females was lesser (9 ± 7 years) than in males (9.3 ± 11.98) which was also statistically insignificant (p = 0.927). According to Le Roy classification, 40 (71.4%) patients belonged to limited variant of SSc (lSSc) while the remaining 16 (28.6%) patients were having the diffuse disease (dSSc).

**Table 1:** **Demographic profile of SSc (N = 56)**

Age – group	Number	(%)
20 - 29	7	12.5%
30 - 39	10	17.9%
40 - 49	12	21.4%
50 - 59	17	30.4%
≥ 60	10	17.9%
Total	56	100%
*Mean ± SD* = 44.96 ± 13.80	*Range =* (21,80)	
**SEX**	**Number**	**%age**
Female	50	89.3%
Male	6	10.7%

Among 31 patients of morphea, females (24; 77.4%) outnumbered the males (7; 22.6%); the average age of the patient was 30.06 ± 10.45 years (14–58). Most of the patients were in the age group 20–29 (11; 35.5%) followed by 30–39 (10; 32.3%) and 10–19 (6; 19.4%) (Table 2). The average age of the onset of the disease in males (24.9 ± 5.96 years) was earlier than in females (28.5 ± 12.42 years) but it was not statistically significant (p = 0.463). Similarly, the mean duration of disease in case of males (2.1 ± 1.76 years) was lesser than in females (2.6 ± 2.34) which was also statistically not significant.

**Table 2:** **Demographic profile of morphea**

Age-group	Number	(%)
10 - 19	6	19.4
20 - 29	11	35.5
30 - 39	10	32.3%
40 - 49	2	6.5%
50 -59	2	6.5%
Total	31	100%
*Mean ± SD* = 30.06 ± 10.45	*Range =* (14,58)	
**SEX**	**Number**	**%age**
Female	24	77.4%
Male	7	22.6%

**Figure 1:**
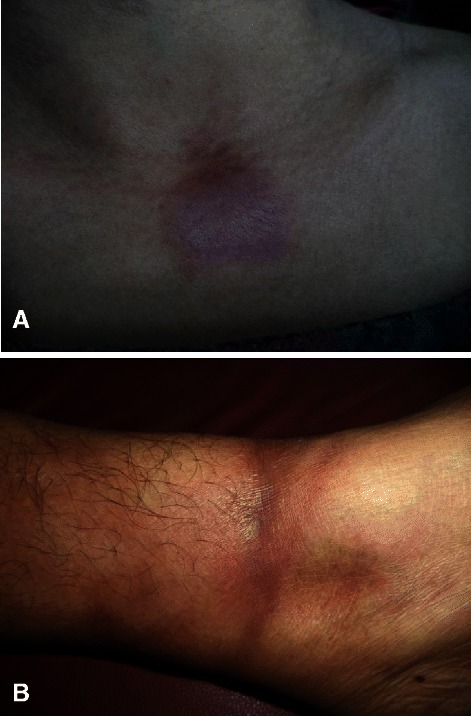
**Clinical types of morphea. A** Plaque type morphea: A brownish hyperpigmented indurated plaque over the upper chest in a 26 year old female. **B** Linear morphea: Brownish hyperpigmented indurated linear plaque encircling lower leg.

The localized plaque type (Figure 1A) morphea was the commonest (21, 67.7%) morphological type seen followed by linear (Figure 1B) (7, 22.6%) and generalized (2, 6.5%) types. Only one (3.2%) patient of morphea Profundus was seen. However, no case of pansclerotic or en coup de sabre was encountered during our study period.

Among 56 patients of SSc, esophageal symptoms (heartburn and/or acid regurgitation and/or dysphagia) were seen in 39 (69.6%) patients; it was mild in 22 (39.3%), moderate in 14 (25%) and severe in 3 (5.3%). On the contrary, only 4 (12.9%) patients of morphea were having esophageal symptoms which were of mild severity and the difference between the two diseases was statistically significant (p < 0.001) (Table 3).

**Table 3:** **Esophageal symptoms in SSc (N=56) and morphea (N=31)**

	Mild symptoms	Moderate symptoms	Severe symptoms	Total symptomatic	P value, significance
Limited SSc (N=40)	15	9	1	25 (62.5%)	0.129 (Not Sig.)
Diffuse SSc (N=16)	7	5	2	14 (87.5%)	
Total SSc	22	14	3	39 (69.6%)	<0.001 (Sig.)
Morphea	4	0	0	04 (12.9%)	

**Figure 2:**
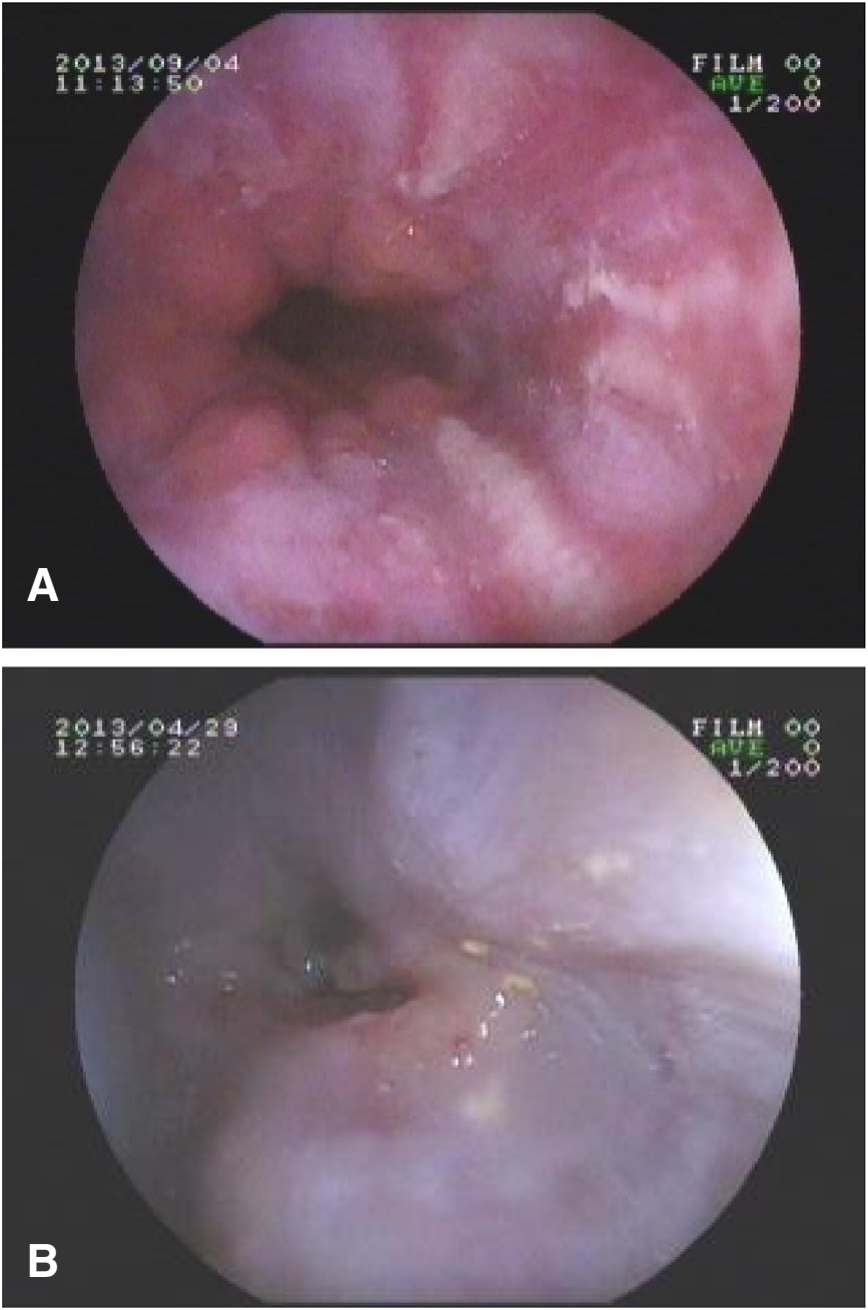
**EGD in systemic sclerosis. A** Grade D esophagitis: Circumferential involvement of lower esophagus involving more than 75% in systemic sclerosis. **B** Esophageal stricture: Narrowed lumen of the lower esophagus due to the longstanding esophagitis in systemic sclerosis.

Reflux esophagitis was seen 17 (32.7%) patients of SSc; it was grade A in 8 (15.4%), grade B in 5 (9.6%), grade C in 2 (3.8%) and grade D (Figure 2A) in only 1 (1.9%).Complicated esophagitis like stricture (Figure 2B) was seen in only 1 (1.9%) patient in our study. Only 2 (7.1%) patients of morphea had esophagitis both of which were of grade A severity and both of them had associated antral gastritis and one of them gave the history of non steroidal anti-inflammatory drug (NSAID) intake (Table 4). It should be noted here that the prevalence of reflux esophagitis in SSc (32.7%) was more than that of morphea (7.1%) and the difference was statistically highly significant (p = 0.022).

**Table 4:** **Reflux esophagitis in SSc and morphea**

Grade	SSc (N=52)		Morphea (N=28)		P –value
	Number	%age	Number	%age	
A	8	15.4	2	7.1	0.022 (Sig.)
B	5	9.6	-	-	
C	2	3.8	-	-	
D	1	1.9	-	-	
Stricture	1	1.9	-	-	
Total Esophagitis	17/52	32.7	2/28	7.1	

Esophageal manometry was studied in 47 and 25 patients of SSc and morphea respectively to look for LES pressure and the contractions of distal body of esophagus. The mean LES pressure was lower (13.2 ± 11.8) in SSc as compared to morphea (31.94 ± 5.61) which was statistically significant (p = <0.001) revealing an overall low LES pressure in SSc patients. Similarly, the mean amplitude of the body of distal esophagus in SSc was less (30.1 ± 29.30) as compared to morphea (77.6 ± 9.38) and the difference was statistically significant (p < 0.001) (Table 5).

**Table 5:** **Esophageal manometry in SSc (N=47) and morphea (N=25)**

Parameter	SSc (Mean, SD)	Morphea (Mean, SD)	P –value	Reference values
LES Pressure	(13.2,11.80)	(31.94, 5.61)	<0.001	10-26 mm Hg
Amplitude of contractions of distal esophagus	(30.1, 29.30)	(77.6, 9.38)	<0.001	50-110 mm Hg

**Figure 3:**
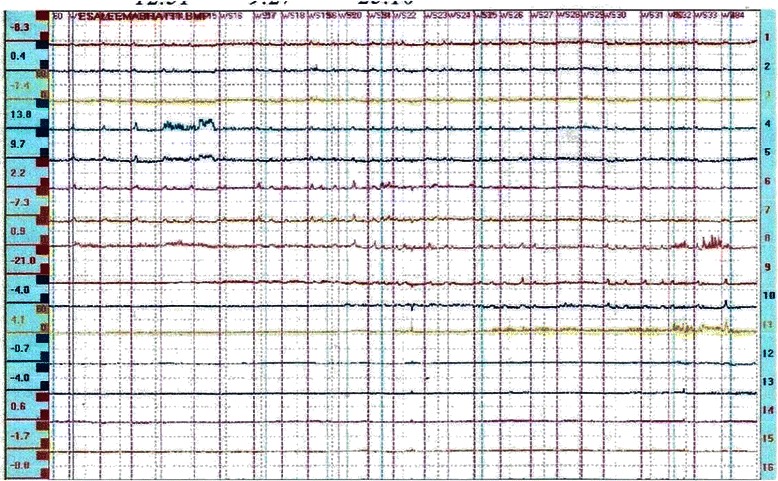
**Esophageal manometry.** Flat waves during esophageal manometry showing aperistalsis in SSc. Note that there are no appreciable esophageal body contractions.

Abnormal manometry was seen in 32 (68.1%) patients of SSc. There was a low LES pressure in 25 (53.2%) patients and distal esophageal body dysmotility in 31 (66%) patients. Out of these 31 patients with esophageal motor disorder (EMD), 19 (40.4%) had hypoperistalsis while the remaining 12 (25.5%) had aperistalsis (Figure 3) (Table 6). On the other hand, none of the patients in the morphea revealed any abnormal manometry.

**Table 6:** **Esophageal manometry in SSc (N=47) and morphea (N=25)**

	SSc		Morphea	
Parameter	Number	%age	Number	%age
Abnormal manometry	32	68.1	0	0
Low LES Pressure	25	53.2	0	0
Esophageal motor disorder(EMD)	31	66.0	0	0
-Hypoperistalsis	19	40.4	0	0
-Aperistalsis	12	25.5	0	0

Forty one patients of SSc and 20 patients of morphea were studied for esophageal pH monitoring. Total reflux time percent, supine reflux time percent, upright reflux time percent and Demeesters score were abnormally high in SSc as compared to morphea patients and the difference was statistically highly significant in each of the four parameters studied. These results showed the significant involvement of esophagus in SSc in comparison to morphea (Table 7).

**Table 7:** **24-hour pH study in SSc and morphea**

Parameter	SSc (Mean, SD)	Morphea (Mean, SD)	P –value	Reference values
Total reflux time percent	(8.5, 4.45)	(1.5, 0.85)	<0.001	<5.5%
Supine reflux time percent	(4.1, 3.80)	(0.6, 0.35)	<0.001	<3%
Upright reflux time percent	(6.1, 5.54)	(2.03, 1.22)	0.002	<8.2%
Demeesters score	(22.9, 13.9)	(3.5, 1.66)	<0.001	<14.7

**Figure 4:**
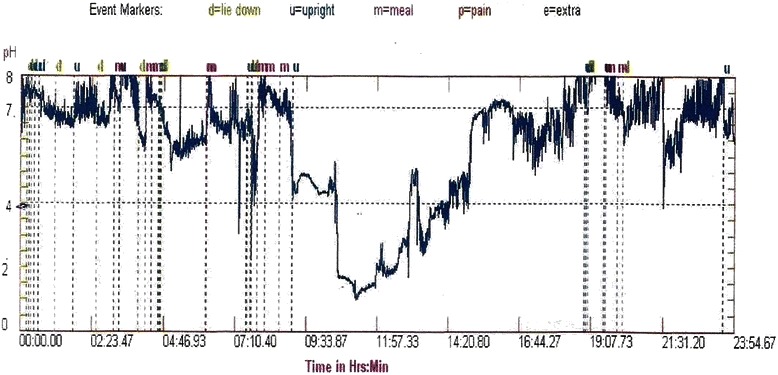
**24-hour pH monitoring.** This is the pH tracing of a systemic sclerosis patient who underwent ambulatory 24-hour pH monitoring. It shows the drop in the pH below 4 and persisting for a longer time (abnormal reflux).

The abnormal reflux (Figure 4) was seen in 33 (80.5%) patients of SSc and they were considered as refluxers. Supine refluxers (15, 45.5%) were the commonest followed by upright (13, 39.4%) and combined refluxers (5, 15.2%). However, in morphea no such abnormal reflux was demonstrated on pH monitoring (Table 8).

**Table 8:** **24-hour pH study results in SSc (N=41) and morphea (N=20)**

Parameter	SSc		Morphea	
	Number	Percent	Number	Percent
Total refluxors	33	80.5	0	0
Upright refluxors	13	39.4	0	0
Supine refluxors	15	45.5	0	0
Combined refluxors	5	15.2	0	0

## Discussion

In our study, females outnumbered males in both SSc and morphea patients which is in accordance with various studies [18,26]. Average age of onset of disease in the SSc patients in our study was earlier in females (fourth decade,35.2 years) than in males which was 42.3 years (5th decade). Similar observations were made by Medsger et al., who studied the epidemiology of SSc and found the peak onset of disease in females in the fourth decade and later in males [26]. The peak incidence of morphea has been estimated to be between 20 and 40 years of age in the literature [27,28]. Similar results were observed in our study as 67.8% of the morphea patients were in the age group 20–39.

Overall incidence of esophageal symptoms in SSc has been estimated between 42% and 79% [29-31]. In our study, the esophageal symptoms were present in 69.6% of the SSc patients which is well in between the range provided by the most published studies. The esophageal involvement in morphea is controversial. Weihrauch et al. studied 14 patients of morphea to assess esophageal involvement by radiography and manometry. Esophageal symptoms were found in only 3 (21.4%) patients [32]. However, in our study the esophageal symptoms were seen in 12.9% of morphea patients. The higher prevalence in the former may be due to their lower sample size. Our study showed a high prevalence of esophageal symptoms in SSc (69.9%) in comparison to the morphea patients (12.9%) which were statistically significant.

Prevalence of reflux esophagitis in SSc has averaged between 30% and 40%. In fact, it is variously reported between 3.2% and 60% [33-37]. Reflux esophagitis in our study, was seen in 32.7% of the patients which is supported by the above studies. Guariso et al. studied 14 patients of morphea for esophageal involvement in a pilot study. He found endoscopically proven esophagitis in 5 (35.7%) patients [38]. However, in our study, only 2 (7.1%) cases of esophagitis were seen revealing a less frequent involvement of esophagus in morphea compared to SSc. Moreover, both these morphea cases that had esophagitis, also had associated antral gastritis; and one of these two patients also gave the history of NSAID intake.

The overall frequency of manometric abnormalities reported in SSc has been very high ranging from 70 - 96%. Reduced LES pressure is present in more than 50% of cases; esophageal motor disorders (EMDs) in more than 60% of cases. Hypoperistalsis has been noted in 48%–81% of cases while aperistalsis in 23%–52% of patients [39]. Lahcene et al. [40] studied the prevalence and risk factors of esophageal motor disorders in systemic sclerosis and found the prevalence of esophageal motor disorders in 81% of patients and a hypotensive lower esophageal sphincter in 62% of the patients. Another study by Savarino, et al. [41] evaluated retrospectively abnormalities of esophageal motility, gastric emptying, oro-cecal transit time (OCTT) and small intestine bacterial overgrowth (SIBO) in a large cohort of SSc patients. Reduced LES pressure and ineffective esophageal motility was encountered in 70% of SSc patients [41]. In our study, the overall manometric abnormalities were seen in 68.1%; low LES pressure in 53.2%; EMDs in 66%; hypoperistalsis in 40.4% and severe aperistalsis in 25.5% cases. All our observations are in accordance with the most published studies. However, none of our morphea patients had any lower esophageal motor abnormalities which are in agreement with the study done by Weirauch et al. Thus, our study revealed a high prevalence of esophageal dysmotility in SSc patients and no such abnormality in morphea patients.

There are many causes of GERD in SSc. The reduction or absence of LES pressure is the primary facilitator of gastric acid reflux into the esophageal lumen. Esophageal dysmotility leads to impaired acid clearance and results in prolongation of esophageal exposure time to gastric acid. Delayed gastric emptying is also a promoter of GERD in SSc patients [42,43]. Currently, Impedance pH-metry is considered to be the gold standard for the diagnosis of GERD [44]. However, impedance pH-metry was not done in our patients as our set up lacked the facility for the same. A study done by Zaninotto et al., showed reflux in 84.6% of the SSc cases; marked abnormalities in esophageal motility and in acid exposure in the distal esophagus were observed in SSc patients only [45]. Another study by Thonhofer et al. [46] investigated the upper GI-tract of patients suffering from SSc and mixed connective tissue disease (MCTD) and found dysmotility of the distal esophagus in 85% of their patients. In our study, abnormal reflux was seen in 80.5% of the cases of SSc. However, not a single case of abnormal reflux was documented in morphea patients. Hence, GERD is significant in SSc only. It should be noted that there were certain limitations in our study. Lack of controls, inability to study upper esophageal sphincter and impedance pH-metry were the limiting factors of the study.

## Conclusion

Esophageal involvement in SSc is very frequent while its involvement in morphea is insignificant. Every patient of SSc needs a meticulous upper gastrointestinal evaluation whether symptomatic or not. However, such an evaluation in morphea seems to be unjustified. It can be inferred that the referral of a SSc patient for EGD, manometry and 24-hour pH study can detect esophageal changes at the earliest and affect the future prognosis of the disease.

## Abbreviations

SSc: Systemic sclerosis
LES: Lower esophageal sphincter
LESP: Lower esophageal sphincter pressure
ARA: American Rheumatology Association
GERD: Gastroesophageal reflux disease
EGD: Esophago-gastroduodenoscopy
SPSS: Statistical package for social sciences
lSSc: Limited SSc
dSSc: Diffuse SSc
NSAID: Non steroidal anti-inflammatory drug
EMD: Esophageal motor disorder
